# Supplementary material for: A minimally sufficient model for rib proximal-distal patterning based on genetic analysis and agent-based simulations
Source: eLife. 2017 Oct 25;6:e29144. doi: 10.7554/eLife.29144 (PMC5693115; doi:10.7554/eLife.29144)
Supplement: Supplementary file 1. [file elife-29144-supp1.pdf]

1 Statistical Analysis of Somite Size and Proliferation

To estimate the size of the somite and the proliferation rate for each genotype, we collected measurements from images taken from somite-matched embryos at E9.0. These measurements can be found in Figure 5 – Source data 1 as an .xlsx file.

To analyze the data, we built a Bayesian hierarchical measurement model using Stan, a probabilistic programming language that allows for statistical modeling (Carpenter et al. 2017). The code is available as Source Code 1 (prolif.R), and Source Code 2 (prolif.stan).

Each data set included groupings of embryos that had control individuals matched with single and/or double combination null embryos whose somites were measured in various ways. For each set or litter, the model estimates a parameter describing the mean size and proliferation rate for the normal condition (parameters named "normsize" and "normscale") as well as a parameter describing the relative size and relative rate for each genotype. These relative sizes and rates are pooled hierarchically to fit a cross-litter distribution whose mean and standard deviation are estimated and the mean of the overall pooled distribution is reported as the overall relative size or relative rate for this genotype. The genotype estimates are "avgGall" and "avgGsizeall". These are the parameters reported in the boxplots of Figure 5.

Various measurements were collected depending on what was possible given the nature of the sample preparation. For example, we were able to collect somite size measurements from samples that had been assayed for other characteristics other than the expression of pHH3.

1. The ratio of the count of cells expressing pHH3 to the count of DAPI positive cells. (proliferation rate)
2. The fraction of total pHH3 positive pixels in a fixed area encompassing much of the somite. (proliferation rate)
3. The ratio of the number of pHH3 cells to the total pixel area of the somite. (proliferation rate)
4. Total DAPI-positive Pixels (size)
5. Total Somite Pixels (size)

Within each set, microscope settings, camera settings, pixel brightness thresholds, were kept constant. However, these settings could vary between sets just due to the nature of doing experiments sequentially. Thus, within each set a reference scale in terms of a control average was estimated. Once a ratio compared to controls was estimated, the ratios are comparable across sets. This allows pooling of the multiple collection points to estimate the genotype-specific average ratio.

These measurements can be on vastly different scales of measurement, but only the relative ratios are of interest as the absolute scales generally reflect for example the image resolution and magnification. We therefore standardized all measurements M by dividing by exp(round(log(median(M)))) which can be thought of as e^N for N an integer exponent relevant for each batch describing the order of magnitude of these measurements. With this rescaling we used gamma(4.0,3.0/1.0) priors on the normscale parameters, reflecting the fact that normal values are O(1) on this scale.

For genotype specific priors we used normal(1.0,1.0) truncated to positive values, reflecting again that the relative size or proliferation rates of the various genotypes are on the order of 1.

The final results reflect all sources of uncertainty including both uncertainty in the genotype-specific measurements and in the control measurements.

Since we plot the parameters as relative ratios, control values are exactly 1.0 by definition.

We then generated Cumulative Distribution Function curves of the genotype specific ratios, to determine the range of credible values for each parameter. We chose the median values for use in our NetLogo simulations.

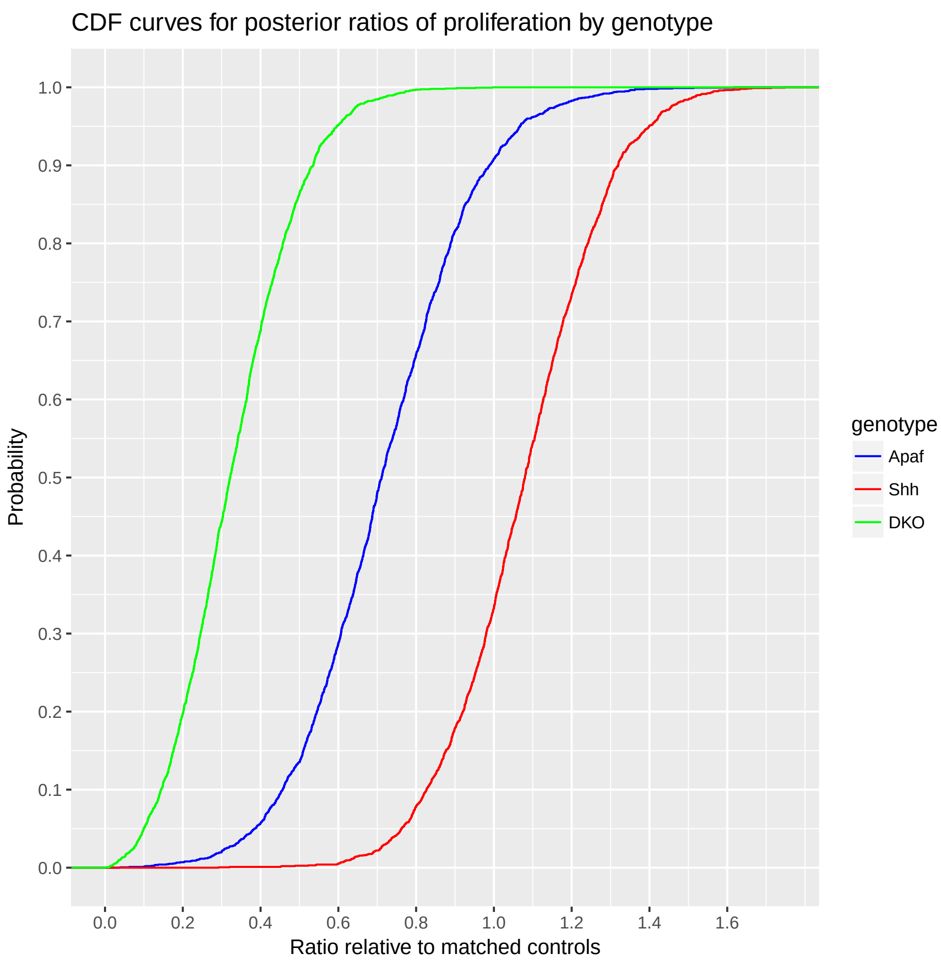
CDF curves for posterior ratios of proliferation by genotype
1.0
0.9
0.8
0.7
0.6
0.5
0.4
0.3
0.2
0.1
0.0
Probability
0.0
0.2
0.4
0.6
0.8
1.0
1.2
1.4
1.6
Ratio relative to matched controls
genotype
Apaf
Shh
DKO

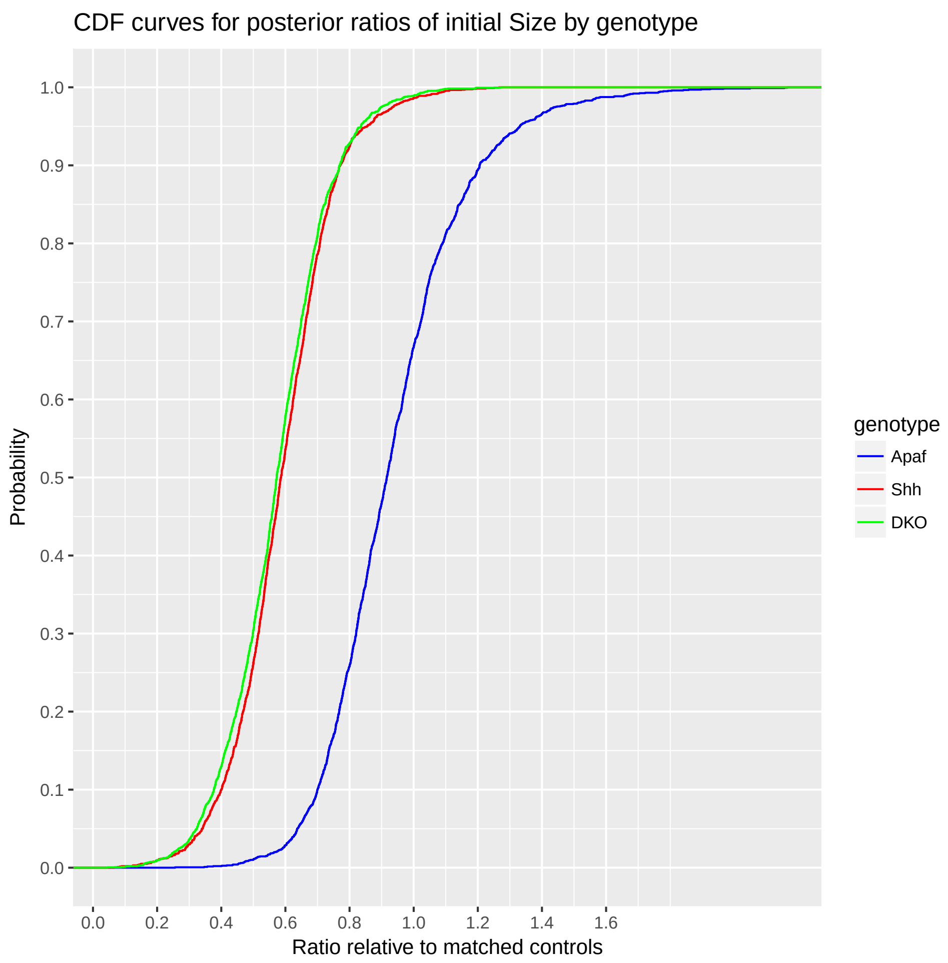

2 References

Carpenter B., Gelman A., Hoffman MD., Lee D., Goodrich B., Betancourt M., Brubaker M., Guo J., Li P., Riddell A. (2017). Stan: A Probabilistic Programming Language. Journal of Statistical Software 76:1, 1-32
