# Supplementary material for: A minimally sufficient model for rib proximal-distal patterning based on genetic analysis and agent-based simulations
Source: eLife. 2017 Oct 25;6:e29144. doi: 10.7554/eLife.29144 (PMC5693115; doi:10.7554/eLife.29144)
Supplement: Supplementary file 2. [file elife-29144-supp2.pdf]

1 Model Description

An agent-based model for rib development was designed in NetLogo to understand the behavior of rib progenitors in the different genetic contexts. NetLogo is a simple freely available software package that allows the user to build simulations of interacting systems of autonomous objects through time (Wilensky, 1999). In NetLogo the objects are called "turtles" and they reside on a two-dimensional field of "patches". Here we use the "turtles" to represent skeletal progenitor cells, and the patches provide a coordinate system that maps to the body wall of the animal. To simulate cellular behavior, we established parameters that could be adjusted depending on the genotype and these are described in the following table.

Variable Name	Description	Purpose
`localfate`	Whether to use neighborhood statistics to determine fate	To investigate the impact of the "community effect" on pattern
`initsizemult`	Sets initial number of agents	To adjust the starting number of agents to account for different initial size conditions.
`shh-xport`	Determines the spatial extent of the shh protein concentration.	Allows the user to adjust the effective diffusivity of the SHH protein
`shh-intensity-log`	Sets the logarithm of the hedgehog protein concentration at x=0.	Allows user to model different protein concentration levels.
`nticks`	Sets the maximum number of time ticks that the simulation will run	Allows the user to adjust the length of developmental time that is simulated.
`pRed1`	A multiplier that adjusts the concentration dependent probability function for deciding cell fate	Allows the user to adjust the effective rate at which cells make decisions to turn red.
`pBlue1`	A multiplier that adjusts the concentration dependent probability function for deciding cell fate	Allows the user to adjust the effective rate at which cells make decisions to turn blue.
`celldeathmult`	A multiplier that adjusts the cell death rate function	To allow the user to adjust the effect of cell death on the simulation.
`prolifratemult`	A multiplier that adjusts the proliferation rate relative to a baseline value.	Allows the user to model the effect of reduced or increased proliferation rate on the simulation.
`cdduration`	A time constant that determines when the cell death rate decays towards zero.	Allows the user to adjust how long cell death occurs.

1.1 Spatial layout

A rectangular two-dimensional field of patches is created with x coordinates in $[-2, 65]$ and y in $[-8, 8]$. Turtles are initially placed in the field so that the left edge of their initial domain is aligned with x=0 and are arranged symmetrically in the y direction around y=0. Patch coordinates are given as integer values, however turtles can occupy any floating point location. For visual reference, each

patch is colored according to the intensity of a continuously varying gradient of the Hedgehog (SHH) protein concentration. This concentration is internally calculated as

$$\mathrm{shh}(x) = I\frac{\exp(-(\frac{(x+10)}{L_s})^2)}{\exp(-(10/L_s)^2)}$$

Here $I = \exp($`shh-intensity-log`$)$ is the intensity multiplier which is adjustable using a slider in the user interface. The function takes on the value I at $x = 0$ and decreases as an inverse quadratic exponential to the right (a normal bell curve) according to a length scale $L_s =$ `shh-xport` also adjustable in the user interface.

To represent a field of embryonic mesenchymal cells, turtles are placed uniformly randomly in a region with left hand x coordinate = 0 and in a square region of constant density of approximately 6 cells per patch. Cells are initialized in an undecided state indicated by the color yellow.

1.2 Dimensionless definition of quantities

All quantities in the model are represented non-dimensionally (as the ratio of two quantities in the physical system).

Accordingly, then, based on the length-scale of the initial field of cells, 1 patch-length represents 1/14 the width of a somite whose initial size multiplier is 1. This is defined as the wild type condition in our simulation.

As time progresses, when `prolifratemult` is set to 1.0 the turtles each have a 0.05 probability of dividing per time tick dt. In our simulations, we define the wild type condition to have `prolifratemult`=1.0. No matter what parameters are chosen in the interface, the time tick $dt = 1$ represents a constant amount of physical time which is $dt = T_2/(\log(2)/\log(1.05)) \approx T_2/14$ where T_2 represents the real doubling time of wild type cells in the embryo in the absence of cell death. To represent other genotypes in which the proliferation rate may be different, the user may set `prolifratemult` to define the proliferation rate as a fraction of the wild type rate of 1.0.

The probability that a cell dies in one tick is $C_d(t)$, a cell death rate function.

$$C_d(t) = 0.05M\exp(-(t/D_d)^2)$$

In this function, D_d is a time-scale for the duration of cell death, `cdduration` in the code, whereas M is a multiplier adjustable in the user interface `celldeathmult`. Cell death is observed to be transient and decay to zero, hence the adjustable decaying function of time. At `celldeathmult` = 1 and `prolifratemult`=1 the initial growth rate would be 0 as proliferation and cell death balance. As the cell death rate function decays the growth rate would increase.

1.3 Tissue Expansion

In order to model expansion of the tissue, we chose to simply enforce a constraint on the turtle density by moving turtles away from regions of high density. Our

expansion algorithm maintains the density of turtles between 4 and 6 per patch. To accomplish this, after the cell-division step, each turtle moves "downhill" in density, away from the direction of the local smoothed density gradient calculated on each patch, with an additional small bias towards expanding outward to the right, as if being dragged by surrounding tissue, plus a diffusive motion occurs, separating cells that are directly on top of each other due to the fact that cells divide in place. At each time tick, this process is iterated until the maximum density of all patches decreases below 6. Turtles do not move unless they occupy a patch with more than 4 turtles, modeling a cohesiveness of cells, and a viscous environment of very low Reynolds number. Therefore, at the end of each time step a near-constant tissue density is maintained. To keep cells from moving outside the boundaries, the code randomly displaces any cells moving through the boundary back into the field. Some small number of turtles are displaced by large amounts early on due to large changes in cell density near the outward moving front. This affects only a tiny fraction of the total turtles, and has no effect on the overall outcome of the simulations.

1.4 Cell Fate

There are three turtle types in the model, represented in the user interface by their color. Yellow colored turtles are undecided, whereas red turtles represent proximal cells and blue turtles represent distal cells. In the code, yellow colored turtles are referred to as "white" but they are colored yellow for better visibility.

At each cell division step, yellow turtles decide whether to divide into two yellow, two red, or two blue turtles. This decision is determined by a random decision-making algorithm taking into account the concentration of the shh(x) function at each turtle location.

To simulate a "community effect", at each patch a statistic describing the redness or blueness of cells on that patch is calculated. The redness and blueness statistics are diffused to smooth them spatially and then if `localfate`=1 these smoothed statistics are used by each turtle to decide whether to switch fates from red to blue or vice versa based on the dominant local color. Fate switching occurs deterministically when a cell finds itself on a patch with its alternate color greater than 60% of the total color value.

NetLogo code does not allow turtles to change their type. Therefore, in our NetLogo code when cells change fate this is implemented by eliminating the previous NetLogo turtle using the `die` function, and creating a new one of the alternative type at the same location.

2 Main algorithm loop

Here we provide a pseudocode version of the main algorithm loop and the tissue expansion method to aid in understanding the actual code, where these functions are implemented by `go` and `move-cells`.

```
while Turtles have not filled the field and time less than max time do
    Have cells divide and/or change colors;
    Update the cell-density, redness, and blueness fields;
    while max cell-density > 6 and no more than 100 steps do
        move cells in opposite direction of density gradient with random
          perturbations to prevent persistent overlapping;
        Update the density, redness, and blueness fields;
    end
    register time tick;
end
```

Algorithm 1: The `go` algorithm

```
foreach turtle do
    if P[x,y] > 4 then
        vx = −(P[x+1,y] − P[x−1,y])/2;
        vy = −(P[x,y+1] − P[x,y−1])/2;
        /* heading has a slight bias to the right, only
           direction not velocity is determined here        */
        set heading (.1+vx),vy;
        /* set velocity based on magnitude of the density
           gradient                                         */
        set v = random uniform(0.5,1) sqrt(vx^2 + vy^2);
        move cell one step;
    end
    adjust turtle location with random normal(0,.5) perturbation to
      smooth motion and separate turtles hatched on top of each other;
    re-satisfy boundaries so no turtles move off field;
end
```

Algorithm 2: the `move-cells` algorithm

Specifics of the mechanisms of these functions are given in the full code (Source Code 3). The full code should be taken as the definitive reference, whereas the pseudocode is to aid in understanding.

3 Simulating the Observed Phenotypes

To model the different phenotypes accurately, we first established values for the normal wild-type situation. The model is defined in terms of dimensionless parameter values that represent the ratio of the actual quantity of the parameter in a given genotype divided by some reference value. For the parameters `initsizemult`, `prolifratemult`, we set normal values to 1 by definition.

For `shh-xport` given that the source was 10 units to the left of the initial field and that the distribution of Sonic Hedgehog has been observed to have a shape similar to a diffusive bell curve (Chamberlain et al., 2008), we chose a value of 12 which allows protein to diffuse a non-trivial distance into the initial field. Based on that choice, we adjusted `shh-intensity-log` to produce a final pattern that was visually approximately 50-50 red-blue, the value was set to 0.6 to represent the wild-type level of Sonic hedgehog, and set to -2.0 when null for *Shh*) at $\exp(-2.6) = 0.074$ of wild-type. We chose this low level (rather than complete absence) to take into account the observation that *Ihh* is ectopically expressed in the absence of *Shh* (Fogel et al., 2008).

Biologically, it is plausible that the wild-type growth of skeletal elements involves an excess capacity which is modulated depending on the growth of the surrounding tissues. For simplicity, because our model involves a constant cell division rate, we have both a set maximum number of time ticks `nticks`, as well as a criterion that stops the clock once enough cells reach the end of the field. Because wild-type is the most robust, we chose our parameter `nticks` equal 46 so that when parameters were set for the *Apaf1* KO (see below) the growth just reaches the end of the frame. Simulations for the wild-type condition stops before this using the early stopping criteria as a simple model of self-modulation. All other genotypes proceed for the full number of time ticks.

We chose pRed1 and pBlue1 so that for the full range of plausible initial sizes, the specification decision would occur while growth remained in the range of morphogen transport. These were sufficiently small such that not every cell division results in a fate choice. The value 0.4 worked reasonably well for all genotypes and the our outcomes were not sensitive to small changes away from this parameter value. The parameter `celldeathmult` which determines the peak intensity of cell death was set to 0.3 for wild-type based on the knowledge that some cell death occurs normally. The duration of cell death, determined by `cdduration` was set to 30, which is a value less than the total growth period of 46 ticks to represent the observation that the cell death period ends prior to full rib outgrowth.

For the other genotypes, the `initsizemult`, `prolifratemult`, and `celldeathmult` values were first chosen by guessing and checking the results of the simulation by eye. In order to match the phenotypes we found that the loss of cell death

in the *Apaf1* KO embryos needed to be compensated by a decrease in initial size and/or proliferation rate in order to match the reduced size of the final structure. We also found that in the *Apaf1;Shh* DKO these reductions needed to be even more severe. Based on this predicted result, we were motivated to measure the size of the somites, as well as the proliferation rate. Using a hierarchical Bayesian measurement error model, we estimated the relative size and relative proliferation rate for each genotype, with the values each normalized to the mean value for wild-type. We then selected the posterior median parameter values for `initsizemult` and `prolifratemult` to generate the simulations, which qualitatively reproduced the final results. See Supplemental File 1 for a description of this statistical model and these exact parameter values.

4 References

Wilensky, U. (1999). NetLogo. http://ccl.northwestern.edu/netlogo/. Center for Connected Learning and Computer-Based Modeling, Northwestern University, Evanston, IL.

Chamberlain CE, Jeong J, Guo C, Allen BL, McMahon AP. Notochord-derived Shh concentrates in close association with the apically positioned basal body in neural target cells and forms a dynamic gradient during neural patterning. Development. 2008 Mar;135(6):1097-106.

Fogel, J. L., Chiang, C., Huang, X. and Agarwala, S. (2008). Ventral specification and perturbed boundary formation in the mouse midbrain in the absence of Hedgehog signaling. Developmental dynamics : an official publication of the American Association of Anatomists 237, 1359-1372.
